# Effects of bioactive glass incorporation into glass ionomer cement on demineralized dentin

**DOI:** 10.1038/s41598-021-86481-y

**Published:** 2021-03-29

**Authors:** Hyun-Jung Kim, Han Eul Bae, Ji-Eun Lee, In-Seong Park, Hee-Gyun Kim, Jiyoung Kwon, Duck-Su Kim

**Affiliations:** 1 Department of Conservative Dentistry, Kyung Hee University Dental Hospital, Seoul, Korea; 2 Department of Conservative Dentistry, Graduate School, Kyung Hee University, Seoul, Korea; 3 Department of Conservative Dentistry, School of Dentistry, Kyung Hee University, 26 Kyungheedae-ro, Dongdaemun-gu, Seoul, 02447 Korea

**Keywords:** Dental materials, Dental biomaterials, Glass-ionomer cement

## Abstract

The effects of the incorporation of sodium-free bioactive glass into glass ionomer cement (GIC) on the demineralized dentin are studied. Four experimental groups with various amounts of BAG in GIC were considered: BG0 group: 0 wt% (control); BG5 group: 5 wt%; BG10 group: 10 wt%; BG20 group: 20 wt%. The GIC surface and GIC-approximated demineralized dentin surfaces were evaluated using field emission scanning electron microscopy (FE–SEM). X-ray diffraction (XRD) analysis was performed to evaluate the chemical changes in the GIC-approximated dentin surface. In addition, a shear bond strength test was performed to evaluate the effects of BAG incorporation on the bond strength of GIC. FE–SEM analysis indicated that BAG-incorporated GICs formed distinct precipitates on their surface. Precipitates were also formed on the GIC-approximated demineralized dentin surface. It was more obvious when the amount of BAG increased. In the XRD analysis, fluorapatitie (FAP) peaks were detected in the BG5, BG10, and BG20 groups. There was no significant difference in the shear bond strength among all experimental groups. BAG-incorporated GIC precipitated FAP crystals underlying demineralized dentin surface without affecting bond strength. This study suggests the possibility of BAG as a beneficial additive in GIC.

## Introduction

Glass ionomer cement (GIC) was developed by Wilson and Kent in 1972. Since then, it has been used widely in dental practices to manufacture dental liners, bases, and restorative and luting agents, and it is recommended for adoption as a restoration material in high-caries-risk lesions. GIC is mainly composed of fluoride-containing alumino-silicate glass powder and polyacrylic acid-based liquid. After the two components are mixed, an acid–base reaction occurs to form a solid mass. GIC has various advantages, such as biocompatibility, tooth adhesion, fluoride release, minimal microleakage, and a coefficient of thermal expansion similar to that of teeth^1–5^. Among them, fluoride release is the most important because it contributes to its anti-cariogenic effects, reduces demineralization, enhances remineralization, and inhibits plaque formation^6–8^.

The acid released during pathological processes (such as erosion or caries progression) or phosphoric acid-etching causes demineralization of dentin^9^. To prevent the cariogenic effect and induce dentin remineralization, fluoride is incorporated into GIC. Fluoride promotes the formation of fluorapatite (FAP) in the presence of calcium and phosphate ions^10^. It inhibits the demineralization of enamel and dentin while enhancing remineralization at crystal surfaces when sufficient calcium and phosphate ions are available^11^. The chemical exchange between GIC and dentin has been proven to be effective in previous study^12^. Therefore, GIC has the potential to inhibit demineralization^13,14^ and promote the remineralization of dentin through the formation of FAP on the adjacent enamel and dentin^15^.

Bioactive glass (BAG) was invented by Dr. Larry Hench in 1969^16^. When BAG is immersed in aqueous solutions, such as artificial saliva (AS) or simulated body fluid (SBF), a rapid cation exchange of Na^+^ and/or Ca^2+^ with H^+^ ions from the solution occurs owing to surface hydrolysis. Phosphate is also leached from BAG. The solution acidity increases gradually, and a silica-rich region forms on the surface of BAG. Soluble silica is lost in the form of Si(OH)_4_ to the solution and repolymerizes in a silica-rich layer. Ca^2+^ and PO_4_^3−^ migrate back from the solution to the surface, forming an amorphous calcium phosphate layer on the silica-rich layer. Finally, hydroxyl ions and carbonate are incorporated from the solution, and calcium phosphate crystallizes to hydroxyapatite (HAP). When BAG contains fluorine or free fluoride ions are available in the surrounding environment, BAG causes the formation of FAP, which is proven to be more resistant to acidic dissolution when compared with HAP^17,18^. Previous studies have indicated that BAG could remineralize enamel^19–21^ and dentin^22,23^. In addition, BAG-containing dental materials, such as adhesives^24^, composite resins^25,26^, and GICs^27–31^, have exhibited the potential to cause dentin remineralization. However, recent studies have evaluated BAG-containing GICs to validate the claims that such a combination will improve tooth bioactivity, regeneration capacity, and restoration^32,33^. Furthermore, there is an ever-increasing interest in the application of bioactive materials in the dental field in an attempt to remineralize the caries-affected dentin^34^.

Based on the results of previous studies, there is a possibility of remineralization synergy if BAG is incorporated into GIC because this material induces coordinated remineralization through the flouride release from GIC and calcium and phosphate supplements from BAG. Choi et al. reported that the addition of BAG to conventional GIC led to the formation of a mineral phase on the surface of GIC, demonstrating the in vitro bioactivity of BAG-incorporated GIC. However, this increases the setting time of GIC^29^. Yli-Urpo et al. reported that the BAG-containing conventional and resin-modified GICs enhanced the in vivo mineralization effect, but negatively affected the compressive strength and surface micro-hardness^27,28,30^. Valanezhad et al. reported that the addition of BAG to resin-modified GIC affected the flexural strength^31,35^ of GIC and reduced cell cytotoxicity^35^.

Although numerous studies on BAG-incorporated GIC have confirmed its bioactivity and ability to change the mechanical properties of GIC, its impact on the adjacent demineralized dentin has not been proven directly. Therefore, this study investigated the effect of BAG-incorporated GIC on demineralized dentin. To enhance the bioactivity of BAG and minimize the adverse effect on the mechanical properties of GIC, a sodium-free, sol–gel derived 63S BAG was used in this study. The null hypothesis of this study is that BAG incorporation into GIC does not affect the bioactivity of GIC.

## Materials and methods

### Materials

Eighty-seven caries-free, human mandibular premolars were used in this study.

The conventional strontium-based GIC, FUJI-IX GP (GC, Tokyo, Japan), was used in this study. Sol–gel derived, sodium-free BAG was purchased from Bonding Chemical (63S BAG; Katy, TX, USA). For specimen storage, AS was prepared and used as per the procedure presented by Garcia-Godoy et al^36^. The materials used in this study are listed in Table 1. There were four experimental groups based on the amount of BAG: BG0 group: 0 wt% (control), BG5 group: 5 wt%, BG10 group: 10 wt%, and BG20 group: 20 wt%. The BAG and GIC powders were mixed for 2 min in a sealed capsule that contained 50 metal balls with a diameter of 2 mm. These groups are summarized in Table 2.

**Table 1:** Composition of materials used in this study.

Materials	Product and manufacturer	Composition
Glass ionomer cement (GIC)	FUJI-IX GP; GC, Tokyo, Japan	Powder: Alumino-fluorosilicate glass and other ingredients Liquid: Polyacrylic acid and proprietary ingredients
Bioactive glass (BAG)	63S BAG; Bonding Chemical, Katy, TX, USA	63% SiO_2_, 31% CaO, 6% P_2_O_5_, > 99%, < 20 μm
Artificial saliva (AS)		CaCl_2_ (0.7 mM/L), MgCl_2_·6H_2_0 (0.2 mM/L), KH_2_PO_4_ (4.0 mM/L) KCl, NaN_3_ (0.3 mM/L), and HEPES buffer (20 mM/L)

**Table 2:** Experimental groups for this study.

Groups	Description
BG0 (control)	Glass ionomer cement (GIC) powder and liquid
BG5	5 wt% BAG incorporated GIC powder and liquid
BG10	10 wt% BAG incorporated GIC powder and liquid
BG20	20 wt% BAG incorporated GIC powder and liquid

### Methods

#### FE–SEM analysis of GIC surface

To evaluate the changes on the surface of each experimental group, experimental GIC powder and liquid were mixed according to the manufacturer’s instructions. The mixed GICs were placed in a silicon mold with a volume of 6 × 6 × 4  mm^3^ to produce blocks. Five blocks were produced for each experimental group and were stored in AS for two weeks. AS was replaced every two days. The blocks were then rinsed with phosphate-buffered saline (PBS) at pH 7.4 for 1 h with three changes followed by rinsing with distilled water for 1 min. The blocks were dehydrated using 25, 50, 75, 95, and 100% ethanol for 20, 20, 20, 30, and 60 min, respectively, and analyzed using field-emission scanning electron microscopy (FE–SEM; Apreo S; Thermo Fisher Scientific, Waltham, MA, USA).

#### FE–SEM analysis of dentin surface

Four experimental groups, namely, BG0, BG5, BG10, and BG20 groups, and one control group (ED group) that evaluated demineralized dentin only were used. Three premolars were used in each group. Three GIC blocks of the same volume (as in “FE–SEM analysis of GIC surface”) were fabricated for each group except for the ED group. Superficial enamel was removed, and the sound dentin surface was exposed to a high-speed diamond saw (Isomet 5000; Buehler Ltd., Lake Bluff, IL, USA). Exposed dentin surfaces were demineralized using a 17% ethylene diamine tetra acetic acid (EDTA) solution (Vista Dental Products, Racine, WI, USA) for 6 h. The GIC blocks were approximated to the demineralized dentin surface as closely as possible. They were fastened using orthodontic bands and stored in AS for two weeks, and AS was changed every two days. After storing, GIC was removed and the dentin surface was washed with deionized water (DW) for 3 min. Dentin specimens were treated as described by Perdigao et al.^37^ The specimens were immediately fixed in a 2.5% glutaraldehyde solution (Sigma-Aldrich, St. Louis, MO, USA) for 12 h. After fixation, the specimens were rinsed with phosphate buffered saline (PBS) for 1 h with three changes followed by rinsing with distilled water for 1 min. The specimens were dehydrated using 25%, 50%, 75%, 95%, and 100% ethanol for 20, 20, 20, 30, and 60 min, respectively. After dehydration, the specimens were immersed in hexamethyldisilazane (Sigma-Aldrich) for 10 min and examined through FE–SEM (Apreo S; Thermo Fisher Scientific).

#### XRD analysis of the dentin surface

X-ray diffraction (XRD) analysis was performed to evaluate the chemical changes in all experimental groups. Three GIC blocks and premolars were processed as described in “FE–SEM analysis of dentin surface” for each experimental group. The dentin surface of each premolar was demineralized using 17% EDTA for 6 h. GIC-approximated dentin specimens were stored in AS for two weeks. After storing, the GIC blocks were removed, and dentin surfaces were rinsed with DW for 3 min. XRD was performed at three different times for the same dentin surface (before demineralization, after demineralization, and after approximation of BAG-containing GIC for two weeks) with an X-ray diffractometer (D8 Advance; Bruker, Billerica, MA, USA) under 40 kV Cu K_α_ radiation using a Ni filter. The diffraction intensities were measured by scanning in the 2*θ* range of 20°–60° in 0.02° steps at a scanning speed of 0.1 s per step.

#### Shear bond strength test

Shear bond strength (SBS) test was performed to evaluate the impact of BAG incorporation on the bond strength of GIC. Fifteen premolars were used in each in experimental group. The superficial enamel was removed, and the sound dentin surface was exposed using a high-speed diamond saw (Isomet 5000). The exposed dentin surfaces were ground using a 600-grit silicon carbide paper to prepare a standard smear layer and were treated with a dentin conditioner (GC) for 20 s to remove the smear layer. Next, the experimental GIC mixture was applied to the dentin surface using an SBS specimen jig (Ultradent Bonding Clamp and Bonding Mold Inserts; Ultradent, South Jordan, UT, USA). After storing in DW for 24 h, SBS was measured using a universal testing machine (AGS-X; Shimadzu, Tokyo, Japan) at a crosshead speed of 1 mm/min.

#### Statistical analysis

The SBS test results were analyzed with one-way analysis of variance (ANOVA) to determine their statistical significance. Tukey’s HSD test was used for *post-hoc* comparison tests. The level of significance was set at *α* = 0.05.

### Ethical standards

#### Ethical approval

The study design was approved by the institutional review board at Kyung Hee University Dental Hospital (KH-DT 19007). All procedures performed in studies involving human participants were in accordance with the ethical standards of the institutional and/or national research committee and with the 1964 Helsinki declaration and its later amendments or comparable ethical standards.

#### Informed consent

For this type of study, formal consent is not required.

#### Institutional review board approval

The IRB of this study was approved by Kyung Hee University Dental Hospital under IRB No. KH-DT19007. In our institution, a comprehensive consent is obtained when a patient’s teeth are extracted in the OMS part. It states that the extracted teeth might be used for research in the future. Therefore, the individual patient’s consent is not required in the studies using only previously extracted teeth.

## Results

### FE–SEM analysis of GIC surface

**Figure 1:**
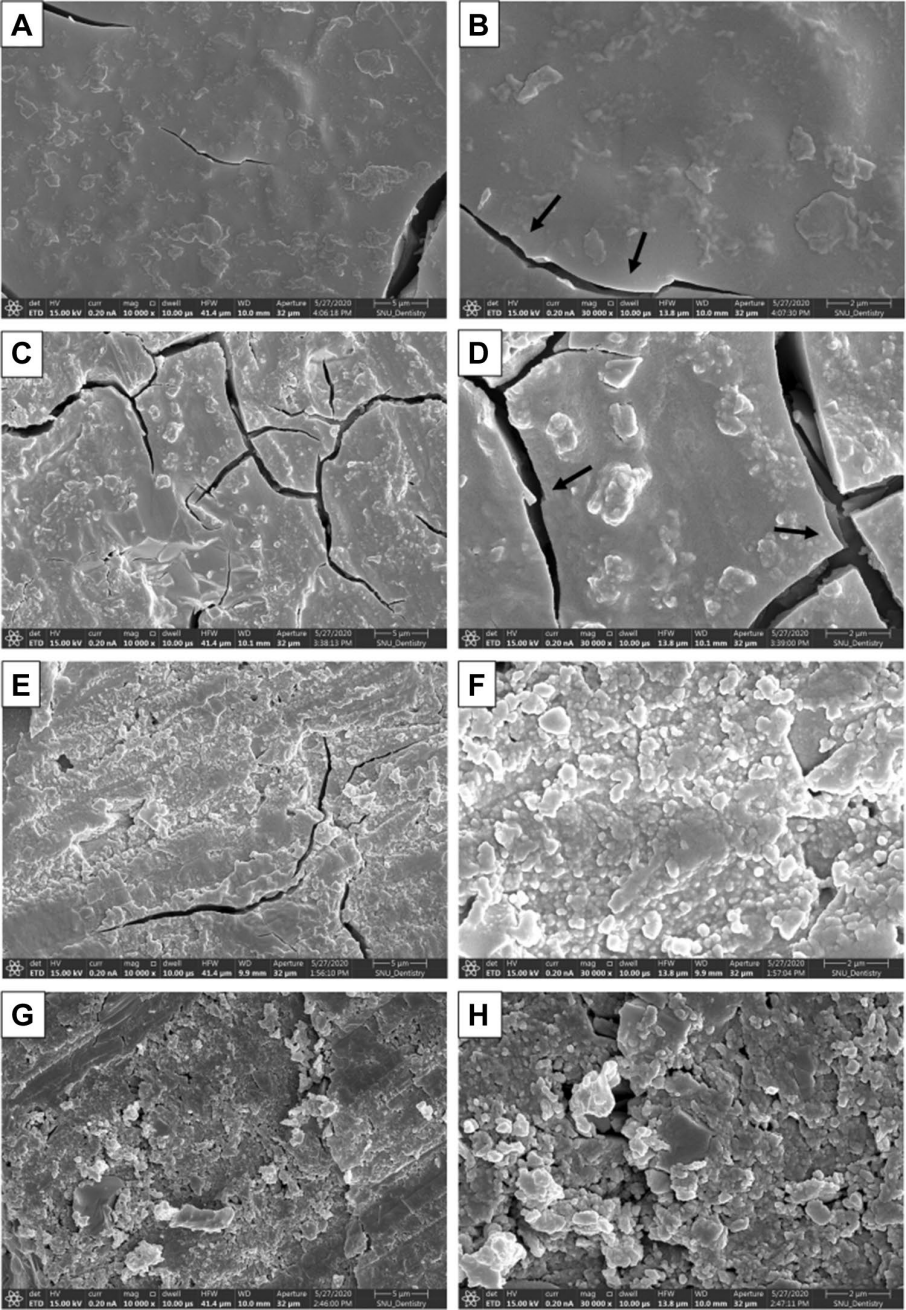
Representative FE–SEM images of GIC surface. (**A**,**B**) BG0 group; unreacted alumino-fluorosilicate glass is observed on the surface. Some crack lines are observed on the surface (black arrow). (**C**,**D**) BG5 group; some surface precipitates as well as crack lines (black arrow) are observed. (**E**,**F**) BG10 group; precipitates formed on the surface, covering the cracks. (**G**,**H**) BG20 group; the observed rough surface of GIC caused by an abundance of precipitate formation. The cracks are hardly observed owing to abundant crystal precipitations.

Figure 1 depicts the GIC surface of all experimental groups. All groups exhibited some cracks on the surface of GIC. There was no surface change in the GIC in the BG0 group (Fig. 1A,B). However, some precipitates were observed on the surface of the GIC in the BG5 group (Fig. 1C,D). These precipitates were more prominent in the BG10 and BG20 groups (Fig. 1E–H).

### FE–SEM analysis of dentin surface

**Figure 2:**
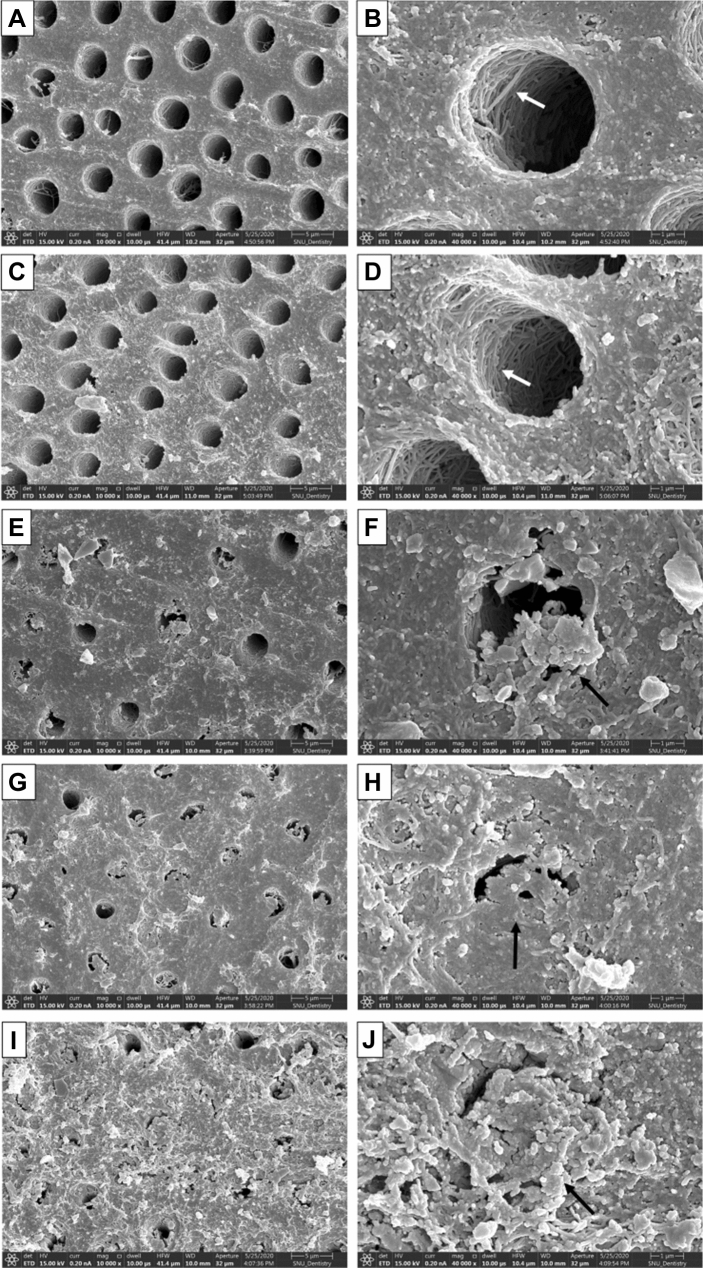
Representative FE–SEM images of dentin surfaces. (**A**,**B**) ED group (control); demineralized, collapsed collagen network is observed. Opened dentinal tubules are clearly seen (white arrow). (**C**,**D**) BG0 Group; demineralized collagen network and opened dentinal tubules are observed (white arrow). (**E**,**F**) BG5 group; some dentinal tubules are partly covered with precipitates (black arrow). (**G**,**H**) BG10 group, some dentinal tubules are almost covered with precipitates (black arrow). (**I**,**J**) BG20 group; most dentinal tubules and dentin surfaces are covered with precipitates (black arrow).

Figure 2 depicts the dentin surfaces of all the experimental groups. In the ED group, the smear layer was removed, and dentinal tubules were almost opened (Fig. 2A,B). Furthermore, collapsed collagen networks were observed on the dentin surface. The FE–SEM images of the BG0 group were similar to those of the ED group (Fig. 2C,D). However, some precipitates were present on the dentin surface and dentinal tubules that were partly covered with precipitates in the BG5 group (Fig. 2E,F). In the BG10 group, more precipitates were observed compared to the BG5 group (Figs. 2G,H). In addition, the dentin surface was almost covered with numerous precipitates in the BG20 group (Fig. 2I,J).

### XRD analysis of dentin surface

**Figure 3:**
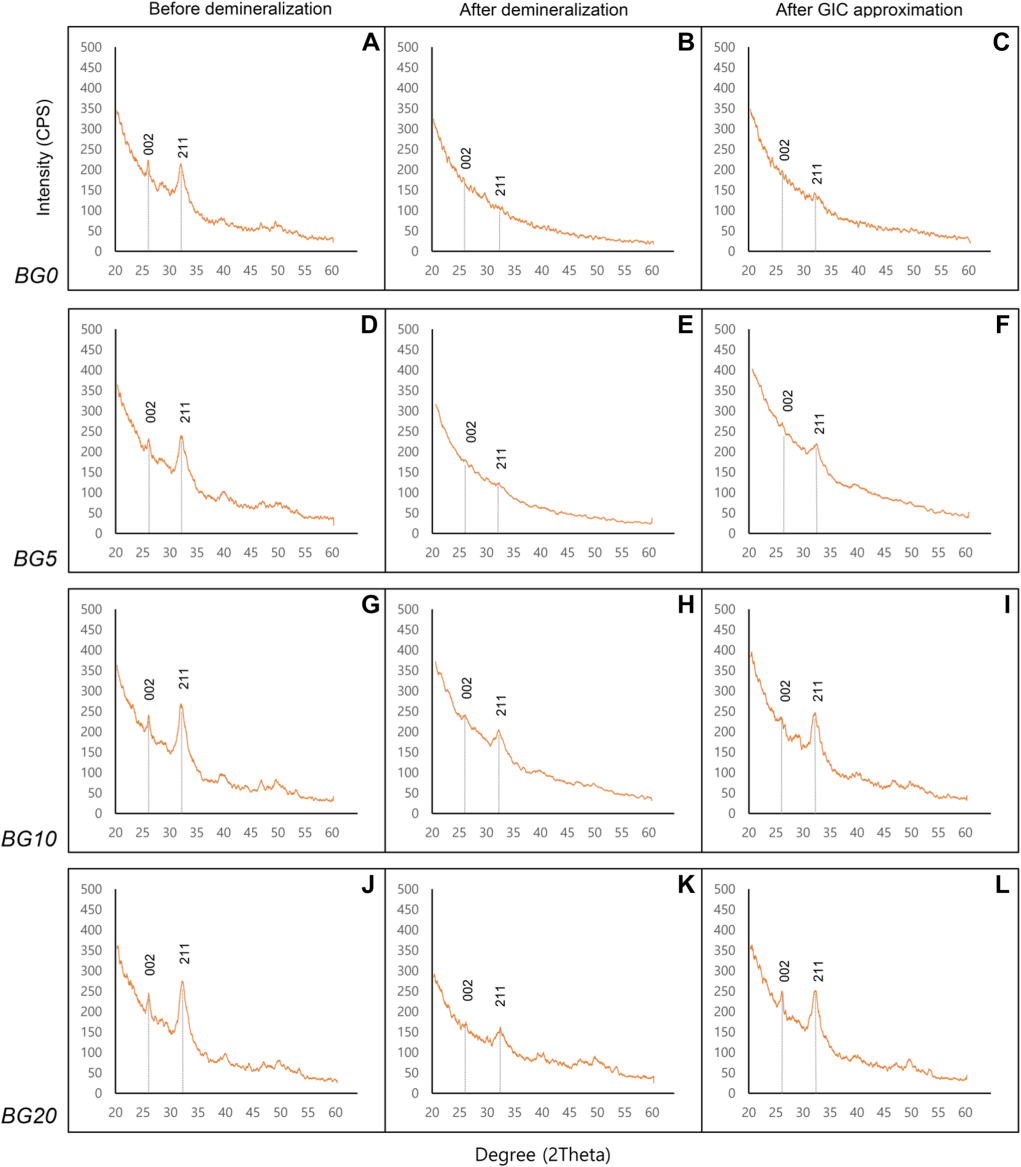
Representative XRD graph of dentin surface. (**A**–**C**) BG0 group. (**D**–**F**) BG5 group. (**G**–**I**) BG10 group. (**J**–**L**) BG20 group. (**A**,**D**,**G**, and **K** are representative of dentin surfaces before demineralization. **B**,**E**,**H**, and **K** are representative of dentin surfaces after demineralization. **C**,**F**,**I**, and **L** are representative of dentin surfaces after GIC approximation). The gray dotted line: 26° (002) and 31.8° (211) peak represent fluorapatite (FAP) or hydroxyapatite (HAP) crystal.

The representative results for the XRD analyses of all the experimental groups are depicted in Fig. 3. There were intense diffraction lines at 26° (002) and 31.8° (211) reflections on the dentin surface before demineralization (Fig. 3A,D,G,J). These peaks are representative of FAP or HAP crystals^38,39^. These exhibited a conspicuous decrease after demineralization (Fig. 3B,E,H,K). In the BG0 group, the peaks rarely increased despite GIC approximation (Fig. 3C). However, the BG5, BG10, and BG20 groups exhibited an increased intensity at both peaks (Fig. 3F,I,L). In particular, the increases in the BG10 and BG20 groups were prominent.

### SBS tests

**Figure 4:**
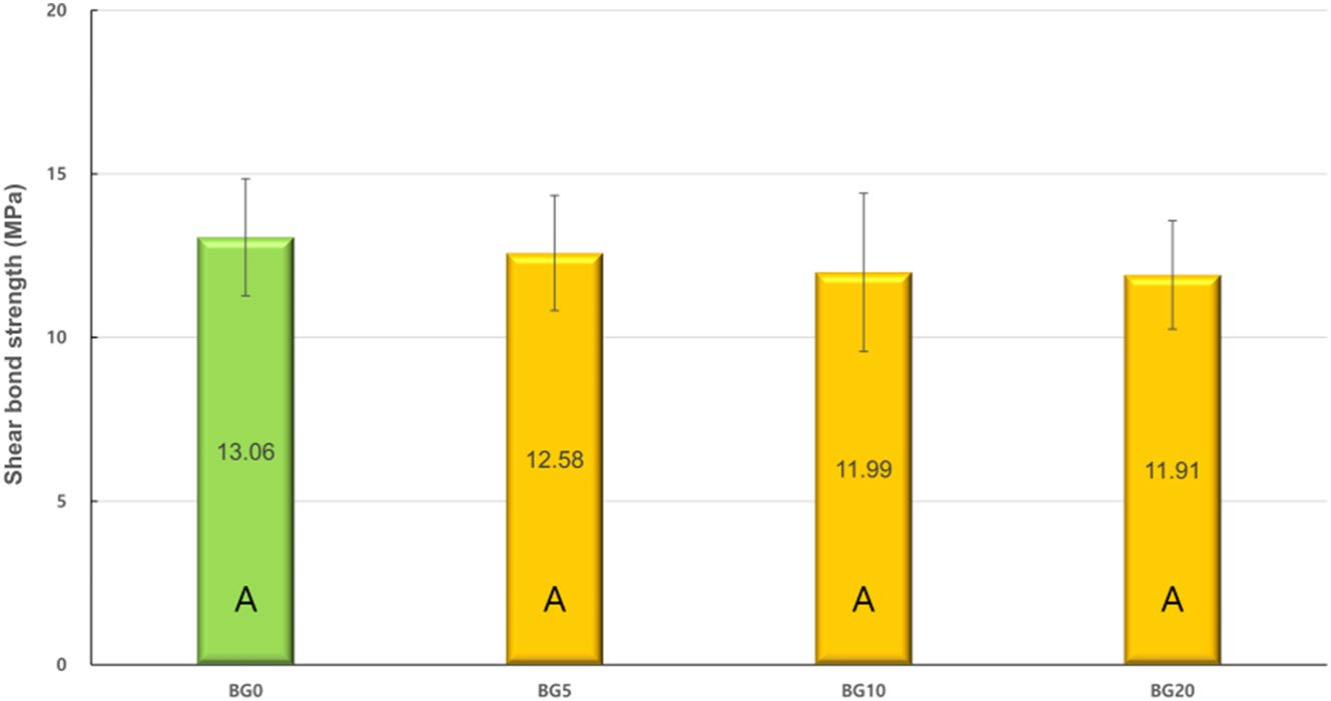
Shear bond strengths of experimental groups. The same capital indicates no statistical difference (*p* > 0.05).

Figure 4 depicts the SBS values of all the experimental groups. The BG0 group exhibited the highest SBS; however, there were no significant differences when compared to those of the BG5, BG10, and BG20 groups (*p* > 0.05).

## Discussion

This study investigated the effects of BAG-incorporated GIC on demineralized dentin. The FE–SEM results indicate that the incorporation of BAG into GIC can induce precipitates on the surface of demineralized dentin. The precipitates were believed to be calcium fluorapatite or strontium fluorapatite on the basis of the XRD analyses. In addition, BAG incorporation did not adversely affect the bond strength of GIC^40,41^.

As described in “Introduction”, GIC has the ability to inhibit demineralization and promote remineralization of teeth owing to the presence of fluoride ions. Fluoride is released from the GIC surface, replaces the hydroxyl ion of HAP, and forms FAP on the surface of teeth, thereby enhancing the resistance against acid attacks. This ability is developed in the presence of adequate levels of calcium and phosphate ions, and the availability of these ions can be a limiting factor for the net tooth remineralization^40^. During the formation of FAP, for every two fluoride ions, ten calcium ions, and six phosphate ions are required to form a unit cell of FAP (Ca_10_(PO_4_)_6_F_2_)^40^. FAP formation is preferred over the production of HAP in a fluoride-containing environment because FAP is chemically more stable and less dissolvable at low pH conditions^18,41^. We believe that the precipitates were FAP even though both crystals have similar peaks in the XRD analysis^42^. In addition, it was also presumed that the strontium-mixed FAP was possibly formed because FUJI-IX GP contains strontium and strontium may be substituted for calcium with virtually no change in the structure^43^. One study revealed that it is impossible to remineralize HAP-depleted dentin^44^. Therefore, we assume that the incorporation of BAG might provide a mineral reservoir and supply calcium and phosphate ions into the interface between GIC and demineralized dentin, which is a prerequisite for crystal precipitation.

In the FE–SEM analysis, all the GIC surfaces exhibited cracks, which may be attributed to dehydration during specimen preparation. In the BG0 and BG5 groups, surface cracks were obvious. The BG10 and BG20 groups exhibited more precipitates in the FE-SEM images (Fig. 1). When the amount of BAG increased in GIC, more precipitates were observed on the dentin surface (Fig. 2). This finding is consistent with those of previous studies^27–29,35^. Yli-Urpo et al. suggested that more calcium ions are present in BAG-containing GIC, as was evidenced by an amorphous calcium phosphate layer^27^. They also reported in vivo mineral deposition around BAG-containing GIC restorations^28^. Choi et al. suggested other evidence for the same finding by weighing their specimens^29^. The XRD analysis confirmed the intense peaks representing FAP or HAP crystals in the BG5, BG10, and BG20 groups (Fig. 3). This means that these crystals are formed on the dentin surface because of GIC incorporation. The precipitates formed on the BAG-containing GIC surfaces were thought to be a calcium phosphate layer in this study. Calcium phosphate layer is regarded as a precursor of HAP, and it can bind the demineralized collagen and form HAP crystals. Notably, peak changes became significantly prominent with an increasing amount of BAG. In addition, it was confirmed that the incorporation of BAG into GIC did not adversely affect the SBS of GIC to dentin in this study (Fig. 4).

Although this study confirmed the benefits of incorporating BAG into GIC, the incorporation of BAG into GIC has some disadvantages also. Yli-Urpo et al. stated that the reason for the decreased mechanical properties of BAG-containing GIC is a loose attachment of BAG particles to the GIC matrix^27^. It could be the formation of sodium polyacrylate salt that inhibits the cross-linking reaction of polyalkenoic acid with Al_3_^+^ and Ca_2_^+^^45^. Therefore, we used a sodium-free BAG for this study to exclude the deteriorating effect of sodium. Based on a study by Choi et al., the setting time for BAG-containing GIC increases proportionally with the amount of BAG^29^. The reason for this is that the cationic release of BAG is lower than that of alumino-fluorosilicate glass, thereby compromising the acid–base reaction of GIC^31^.

The viscosity of GIC is a crucial factor because it can hinder the clinical application of GIC in oral cavities. In this study, the viscosity of BAG-incorporated GIC tended to increase with an increase in the amount of BAG. This increase in viscosity may be related to silica, which is one of the main components of BAG. Silica tends to self-agglomerate and can increase the viscosity of the GIC mixture. For clinical use and commercialization of the BAG-incorporated GIC, it is necessary to determine the optimum ratio of the BAG to GIC powder, which can satisfy both the remineralization capacity and handling properties.

It is not easy to evaluate the morphological changes on the dentin surface when GIC is bonded. Therefore, GIC blocks were approximated to demineralized dentin surfaces in this study. This method has been used in a previous study and proven to be effective^25^. AS was used as the storage solution in this study, although SBF can also be used. However, it may cause an autogenous precipitation of calcium phosphates on the surface of dentin. In this study, dentin demineralization was performed with 17% EDTA because it is a strong chelating agent for dentin and can simulate harsh, mineral-depleted dentin surfaces. According to Kawasaki et al., EDTA rarely leaves minerals on the surface of dentin^46^. The FE–SEM analysis of the ED and BG0 groups also indicated collapsed collagen networks on the dentin surface (Fig. 2B,D). Vollenweider et al. reported that the demineralized dentin with EDTA might cause this situation in addition to the lack of nuclei on the dentin surface^23^. Despite this harsh environment, BAG-incorporated GIC induced remarkable apatite crystal precipitation on the demineralized dentin surface in this study. The FE–SEM analyses of the BG5, BG10, and BG20 groups indicated some precipitates that partly covered the dentin surface. The presence of the precipitate was more prominent when the amount of BAG increased. This phenomenon was similar to that of the GIC surface.

The initial acidity of the GIC setting reaction, involving a prolonged period at pH below 3, affects not only the pH of the setting cement but also the quantity of the available acid at the dentin interface^47^. In the first reaction stage, BAG can uptake H^+^ or H_3_O^+^ ions from the solution, causing hydrolysis of the silica groups and enabling the release of calcium and phosphate ions^48^. The pH of the solution increases as result of H^+^ ions in the solution being replaced by cations. The pH changes in the BAG reaction might increase the pH of the GIC setting environment. One study suggests that the GIC-BAG composite increases the pH value in an aqueous environment^49^. The increased pH may affect the mineralizing property of the BAG-incorporated GIC and impart an antimicrobial effect to it.

XRD analyses were performed to evaluate the chemical changes on the dentin surface with or without the approximation of BAG-incorporated GIC. The present study differs from the previous ones in that it evaluated the same tooth at three different times: before demineralization, after demineralization, and after approximation of BAG-incorporated GIC for two weeks. The reason for using the same tooth was that the histological variations in different teeth might result in analytical differences. Although no statistical analysis was not performed, the BG10 and BG20 groups indicated an increased FAP peaks after the approximation. This was consistent with the results of the FE–SEM analysis.

SBS test was performed to evaluate the effects of BAG incorporation on GIC adhesion. Although the micro-tensile bond strength test is used at present, it is not an appropriate method to evaluate the low bond strength of GIC. In this study, a dentin conditioner was used to increase the bond strength and minimize the pre-testing failure. The dentin conditioner contains a diluted solution of polyacrylic acid and removes superficial smear layers on the surface of the dentin, thereby improving chemical adhesion^50^. Ionic bond, between the calcium ions of HAP or FAP and the carboxylate groups of GIC, is the mechanism through which GIC adheres to the dentin^51^. In this study, BAG incorporation did not adversely affect the SBS of GIC regardless of the amount of BAG, indicating that BAG did not affect the adhesion mechanism of GIC. The use of sodium-free BAG was required to maintain the SBS of the BAG-incorporated GIC.

This study was designed to evaluate the effects of BAG-incorporated GIC on demineralized dentin. Based on the results of this study, BAG-incorporated GIC exhibited the ability to induce crystal precipitation on demineralized dentin. Further studies are necessary to support the results of this study and investigate the changes in mechanical properties on the dentin surface and long-term effect of BAG-incorporated GIC.

## Conclusion

Within the limitation of this study, we conclude that BAG-incorporated GIC could precipitate FAP crystals underlying demineralized dentin without hampering the bond strength. BAG is an advantageous supplement to GIC, and its incorporation up to 20 wt% is beneficial for FAP crystal precipitation of demineralized dentin. This study suggests the possibility of using BAG as a functional additive in GIC for clinical settings.

## References

[1] Forss H, Jokinen J, Spets-Happonen S, Seppa L, Luoma H (1991). Fluoride and mutans streptococci in plaque grown on glass ionomer and composite. Caries Res..

[2] Preston AJ, Mair LH, Agalamanyi EA, Higham SM (1999). Fluoride release from aesthetic dental materials. J. Oral Rehabil..

[3] Tyas, M. J. & Burrow, M. F. Adhesive restorative materials: a review. *Aust. Dent. J.* **49,** 112–121 (quiz 154) (2004). 10.1111/j.1834-7819.2004.tb00059.x 15497354

[4] Nicholson JW (1998). Chemistry of glass-ionomer cements: a review. Biomaterials.

[5] Nicholson JW (2007). Polyacid-modified composite resins ("compomers") and their use in clinical dentistry. Dent. Mater..

[6] Komatsu H, Shimokobe H, Kawakami S, Yoshimura M (1994). Caries-preventive effect of glass ionomer sealant reapplication: Study presents three-year results. J. Am. Dent. Assoc..

[7] Mukai M (1993). Fluoride uptake in human dentine from glass-ionomer cement in vivo. Arch. Oral Biol..

[8] Retief DH, Bradley EL, Denton JC, Switzer P (1984). Enamel and cementum fluoride uptake from a glass ionomer cement. Caries Res..

[9] Rodríguez-Sendra J, Torres I, Jiménez N, Sauro S, Camarena F (2020). Ultrasonic monitoring of dentin demineralization. IEEE Trans. Ultrason. Ferroelectr. Freq. Control.

[10] Hemagaran G, Neelakantan P (2014). Remineralization of the tooth structure-the future of dentistry. Int. J. Pharmtechnol. Res..

[11] Featherstone J, Glena R, Shariati M, Shields C (1990). Dependence of in vitro demineralization of apatite and remineralization of dental enamel on fluoride concentration. J. Dent. Res..

[12] Ngo HC, Mount G, Mc Intyre J, Tuisuva J, Von Doussa RJ (2006). Chemical exchange between glass-ionomer restorations and residual carious dentine in permanent molars: an in vivo study. J. Dent..

[13] Dionysopoulos P, Kotsanos N, Koliniotou-Koubia E, Tolidis K (2003). Inhibition of demineralization in vitro around fluoride releasing materials. J. Oral Rehabil..

[14] Francci C (1999). Fluoride release from restorative materials and its effects on dentin demineralization. J. Dent. Res..

[15] ten Cate JM, van Duinen RN (1995). Hypermineralization of dentinal lesions adjacent to glass-ionomer cement restorations. J. Dent. Res..

[16] Jones JR (2013). Review of bioactive glass: From Hench to hybrids. Acta Biomater..

[17] Brauer DS, Karpukhina N, O’Donnell MD, Law RV, Hill RG (2010). Fluoride-containing bioactive glasses: Effect of glass design and structure on degradation, pH and apatite formation in simulated body fluid. Acta Biomater..

[18] Mneimne M, Hill RG, Bushby AJ, Brauer DS (2011). High phosphate content significantly increases apatite formation of fluoride-containing bioactive glasses. Acta Biomater..

[19] Farooq I, Majeed A, AlShwaimi E, Almas K (2019). Efficacy of a novel fluoride containing bioactive glass based dentifrice in remineralizing artificially induced demineralization in human enamel. Fluoride.

[20] Kanwal N (2018). In-vitro apatite formation capacity of a bioactive glass-containing toothpaste. J. Dent..

[21] Bakry AS (2018). A novel fluoride containing bioactive glass paste is capable of re-mineralizing early caries lesions. Materials.

[22] Jung JH (2019). Dentin sealing and antibacterial effects of silver-doped bioactive glass/mesoporous silica nanocomposite: An in vitro study. Clin. Oral Investig..

[23] Vollenweider M (2007). Remineralization of human dentin using ultrafine bioactive glass particles. Acta Biomater..

[24] Jun SK (2018). Multi-functional nano-adhesive releasing therapeutic ions for MMP-deactivation and remineralization. Sci. Rep..

[25] Jang JH (2018). Effect of bioactive glass-containing resin composite on dentin remineralization. J. Dent..

[26] Tezvergil-Mutluay A (2017). Effects of composites containing bioactive glasses on demineralized dentin. J. Dent. Res..

[27] Yli-Urpo H, Lassila LV, Narhi T, Vallittu PK (2005). Compressive strength and surface characterization of glass ionomer cements modified by particles of bioactive glass. Dent. Mater..

[28] Yli-Urpo H, Narhi M, Narhi T (2005). Compound changes and tooth mineralization effects of glass ionomer cements containing bioactive glass (S53P4), an in vivo study. Biomaterials.

[29] Choi JY, Lee HH, Kim HW (2008). Bioactive sol-gel glass added ionomer cement for the regeneration of tooth structure. J. Mater. Sci. Mater. Med..

[30] Khoroushi M, Mousavinasab SM, Keshani F, Hashemi S (2013). Effect of resin-modified glass ionomer containing bioactive glass on the flexural strength and morphology of demineralized dentin. Oper. Dent..

[31] Kim DA (2017). Sol-gel-derived bioactive glass nanoparticle-incorporated glass ionomer cement with or without chitosan for enhanced mechanical and biomineralization properties. Dent. Mater..

[32] Yli-Urpo H, Närhi M, Närhi T (2005). Compound changes and tooth mineralization effects of glass ionomer cements containing bioactive glass (S53P4), an in vivo study. Biomaterials.

[33] Ana ID, Matsuya S, Ohta M, Ishikawa K (2003). Effects of added bioactive glass on the setting and mechanical properties of resin-modified glass ionomer cement. Biomaterials.

[34] Khoroushi M, Keshani F (2013). A review of glass-ionomers: From conventional glass-ionomer to bioactive glass-ionomer. Dent. Res. J..

[35] Valanezhad A (2016). Modification of resin modified glass ionomer cement by addition of bioactive glass nanoparticles. J Mater. Sci. Mater. Med..

[36] Garcia-Godoy F (2007). Degradation of resin-bonded human dentin after 3 years of storage. Am. J. Dent..

[37] Perdigao J, Lambrechts P, Van Meerbeek B, Vanherle G, Lopes AL (1995). Field emission SEM comparison of four postfixation drying techniques for human dentin. J. Biomed. Mater. Res..

[38] Brundavanam RK, Poinern GEJ, Fawcett D (2013). Modelling the crystal structure of a 30 nm sized particle based hydroxyapatite powder synthesised under the influence of ultrasound irradiation from x-ray powder diffraction data. Am. J. Mater. Sci..

[39] Aljabo A, Abou Neel EA, Knowles JC, Young AM (2016). Development of dental composites with reactive fillers that promote precipitation of antibacterial-hydroxyapatite layers. Mater. Sci. Eng. C.

[40] Reynolds EC (2008). Calcium phosphate-based remineralization systems: scientific evidence?. Aust. Dent. J..

[41] Shah FA (2016). Fluoride-containing bioactive glasses: Glass design, structure, bioactivity, cellular interactions, and recent developments. Mater. Sci. Eng. C.

[42] Zhao J (2014). Solution combustion method for synthesis of nanostructured hydroxyapatite, fluorapatite and chlorapatite. Appl. Surf. Sci..

[43] Stamboulis A, Law RV, Hill RG (2004). Characterisation of commercial ionomer glasses using magic angle nuclear magnetic resonance (MAS-NMR). Biomaterials.

[44] Kim YK (2010). Failure of a glass ionomer to remineralize apatite-depleted dentin. J. Dent. Res..

[45] De Barra E, Hill R (1998). Influence of alkali metal ions on the fracture properties of glass polyalkenoate (ionomer) cements. Biomaterials.

[46] Kawasaki K, Ruben J, Tsuda H, Huysmans M, Takagi O (2000). Relationship between mineral distributions in dentine lesions and subsequent remineralization in vitro. Caries Res..

[47] Smith DC, Ruse ND (1986). Acidity of glass ionomer cements during setting and its relation to pulp sensitivity. J. Am. Dent. Assoc..

[48] Rabiee SM, Nazparvar N, Azizian M, Vashaee D, Tayebi L (2015). Effect of ion substitution on properties of bioactive glasses: A review. Ceram. Int..

[49] Yli-Urpo H, Söderling E, Vallittu PK, Närhi T (2004). pH changes induced by bioactive glass ionomer cements. Key Eng. Mater..

[50] Powis DR, Follerås T, Merson SA, Wilson AD (1982). Improved adhesion of a glass ionomer cement to dentin and enamel. J. Dent. Res..

[51] Yoshida Y (2000). Evidence of chemical bonding at biomaterial-hard tissue interfaces. J. Dent. Res..

